# 1898. Optimizing selection of latent TB management strategies among immigrants from high TB burden settings to the United States

**DOI:** 10.1093/ofid/ofad500.1726

**Published:** 2023-11-27

**Authors:** Pranay Sinha, Karen R Jacobson, C Robert Horsburgh, Benjamin P Linas, Carlos Acuña-Villaorduña

**Affiliations:** Boston University, Boston, Massachusetts; Boston University School of Medicine, Boston, Massachusetts; Boston University, Boston, Massachusetts; Boston University School of Medicine/Boston Medical Center, Boston, MA; Boston University School of Medicine, Boston, Massachusetts

## Abstract

**Background:**

Treatment for latent tuberculosis infection (LTBI) with rifampin and isoniazid is very effective but carries significant hepatotoxicity risks, increasing with age. Further, since the risk of progression to tuberculosis (TB) disease declines with increasing time from exposure, the risk is highest among recent immigrants. Current guidelines may therefore promote overtreatment of LTBI, particular in older immunocompetent individuals who immigrated many years previously.

**Methods:**

Using observed data from the Boston Medical Center/Boston Public Health Commission TB clinic and best literature estimates, we designed a microsimulation model to simulate TB progression among immunocompetent adult immigrants from high TB burden countries without abnormal radiography, alcohol or tobacco use, or recent TB contacts over a 30-year horizon (Table 1). We assumed that the simulated individuals were exposed to TB immediately prior to immigration. TB progression rates are based on years since exposure (Table 1). We evaluated three strategies: 1. No treatment 2. Rifampin and 3. Isoniazid for new 35 years-old and 65 years-old immigrants and for 35 and 65 years-old immigrants who had immigrated 25 years previously. We simulated hepatotoxicity using published estimates (Table 2). The model calculates costs and outcomes (TB cases and disability-adjusted life years) associated with each strategy. We used deterministic and probabilistic sensitivity analyses to test robustness of our results.

**Results:**

Among new immigrants both 35 and 65 years-old, rifampin provides better outcomes at lower cost than both the isoniazid and no treatment strategies (Table 3). Among those who immigrated 25 years ago, Rifampin remains the preferred strategy in 75% of simulations for 35 years-old individuals and no treatment was preferred in 100% of iterations for 65 years-old patients. For those unable to receive rifampin due to drug interactions, no treatment became the preferred strategy for a broader range of individuals (Figures 1a and 1b).

**Conclusion:**

Age and time since immigration must be considered to determine whether LTBI therapy is indicated in immunocompetent immigrants from high TB burden countries. Our results could help clinicians optimize strategies for LTBI treatment and minimize harm.

**Disclosures:**

**All Authors**: No reported disclosures

